# Killer-cell Immunoglobulin-like Receptor gene linkage and copy number variation analysis by droplet digital PCR

**DOI:** 10.1186/gm537

**Published:** 2014-03-05

**Authors:** Chrissy h Roberts, Wei Jiang, Jyothi Jayaraman, John Trowsdale, Martin J Holland, James A Traherne

**Affiliations:** 1 London School of Hygiene and Tropical Medicine, Keppel St, London WC1E 7HT, UK; 2 Cambridge Institute for Medical Research, Addenbrooke’s Hospital, Cambridge CB2 0XY, UK; 3 Division of Immunology, Department of Pathology, University of Cambridge, Cambridge CB2 1QP, UK

## Abstract

The Killer-cell Immunoglobulin-like Receptor (KIR) gene complex has considerable biomedical importance. Patterns of polymorphism in the KIR region include variability in the gene content of haplotypes and diverse structural arrangements. Droplet digital PCR (ddPCR) was used to identify different haplotype motifs and to enumerate KIR copy number variants (CNVs). ddPCR detected a variety of KIR haplotype configurations in DNA from well-characterized cell lines. Mendelian segregation of ddPCR-estimated *KIR2DL5* CNVs was observed in Gambian families and CNV typing of other KIRs was shown to be accurate when compared to an established quantitative PCR method.

## Background

**Figure 1:**
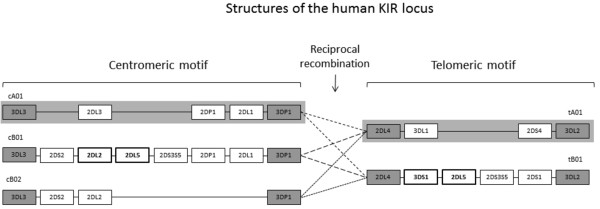
**Organization of the KIR locus on the human chromosome region 19q13.4.** KIR haplotypes are composed of different centromeric (cA01, cB01 and cB02) and telomeric motifs (tA01, tB01) [10]. Each motif has a different content and arrangement of genes. Framework genes, which are found at the ends and near the middle of the locus on nearly all haplotypes [11] are shaded grey. The *KIR2DL5* gene can be located in either the centromeric or telomeric motif, adjacent to *KIR2DL2* and *KIR3DS1*, respectively, or on both motifs of a single haplotype. Different centromeric motifs can be paired with different telomeric motifs through the central reciprocal recombination hotspot between *KIR3DP1* and *KIR2DL4*, as indicated by different dashed lines. KIR haplotypes can be classified into two categories [12]: group A haplotypes (shaded grey), composed of cA01 and tA01 motifs only, have fixed gene-content with one activating gene (*KIR2DS4*); group B haplotypes (unshaded), comprising at least one motif of type cB01, cB02 or tB01, have variable gene content between framework genes and more than one activating KIR locus.

A highly polymorphic gene complex on chromosome 19q13.4 encodes the Killer-cell Immunoglobulin-like Receptors (KIRs) [1]. As natural killer (NK) cell receptors, their roles in modulating outcomes of infectious diseases [2,3], transplantation [4] and pregnancy [5] are subjects of intense research, but their genotyping and subsequent use in association studies and histocompatibility matching are made difficult by the underlying structural and sequence complexity in the KIR region. Both haplotype structural variations [6] and KIR gene copy number variations (CNVs) [7,8] contribute to the diversity of the KIR system. The highly homologous KIR genes are arranged closely head-to-tail, an arrangement that facilitates non-reciprocal recombination (misalignment and unequal crossovers during meiosis), which can delete, duplicate or recombine genes [9] (Figure 1). The implications of this are that KIR genes may be present or absent from a haplotype, that those KIR genes that are present on a haplotype may occur multiple times, and that the structural arrangement of the KIR genes may vary between individuals with otherwise identical gene contents and/or gene copy numbers.

The KIR complex is divisible into two variable motifs that are defined by their orientation towards the centromeric (Cen) or telomeric (Tel) regions of the chromosome [13]. *KIR2DL5* was the first KIR gene that was widely recognized as segregating to more than one locus [14,15] and is common in most human populations. At the *KIR2DL5C* locus, *KIR2DL5* is often (though not exclusively) found to be adjacent to *KIR2DL2*[13]. At the *KIR2DL5T* locus, *KIR2DL5* is frequently found to be adjacent to *KIR3DS1*[13]. In some haplotypes, *KIR2DL5* is present, whilst both *KIR2DL2* and *KIR3DS1* are absent [16]. An individual haplotype might have zero (Figure 1, cA01 ~ tA01 conformation), one (Figure 1, cB01 ~ tA01, cA01 ~ tB01 and cB02 ~ tB01 conformations), or two (Figure 1, cB01 ~ tB01 conformation) copies of *KIR2DL5* (EMBL accession AY320039; Gassner, C., Williams, L.M., Yamashita, T., Selvakumar, A., Dupont, B. and Geraghty, D.E., unpublished data) and might possess either, both, or neither of the *KIR2DL2* ~ *KIR2DL5C* and *KIR3DS1* ~ *KIR2DL5T* gene arrangements (Figure 1). Consequently, an individual might have up to four copies of *KIR2DL5.*

Detailed characterization of KIR haplotypes is possible using a combination of pedigree analyses, high-resolution molecular typing and likelihood estimations [17,18]. These approaches are costly, not easily scalable to high throughputs and are frequently not completely accurate. All current methods rely, at least in part, on the specification *a priori* of known haplotype structures*.* These haplotype maps are derived from a small number of fully sequenced KIR haplotypes [13,17,19] and also from observed patterns of perfect linkage disequilibrium between key pairs of KIR genes [10,15,18,20,21]. These assumptions appear to hold true in most Caucasian populations [10,17,20-22] but may be invalid in the wider global population, especially in African populations [23]. Empirical approaches to the definition of haplotype structural diversity in global populations are required, but options are currently limited to pedigree analysis [20,21] and sequencing of single chromosomes [11,24,25]. Molecular haplotyping is possible in single-molecule processes, including digital PCR [26-30], and recent reports have highlighted how CNVs can be enumerated using droplet digital PCR (ddPCR) [31,32].

In this application of digital PCR, a limiting number of DNA target molecules are stochastically confined by a microfluidic device [33] into a large number of droplet PCR nano-reactors (volume 10^-9^ L) that contain either zero or one copy of the PCR target. The ddPCR reaction may be a duplex test that simultaneously detects two targets using fluorescent probes. After PCR is complete, the droplets are passed in single-file through a flow cytometric device, which determines the qualitative end-points of PCR by assaying the presence or absence of hydrolysis probe-derived fluorescence signals. Counts of PCR-positive and PCR-negative droplets are made and these are converted into an accurate measure of the number of target entities (copies/volume) in the total PCR volume without the need to refer to calibration curves or reference samples [34-36].

When there are two ddPCR targets that are not physically linked (either because they originate on different chromosomes or if intra-chromosomal linkage has been cut or sheared during or subsequent to DNA extraction), then two independent stochastic DNA confinement processes occur and these may overlap. In the absence of linkage, the result is that a droplet may contain zero, one, or both targets. In the presence of linkage, the confinement processes are not independent and the frequency of 'double positive' droplets is substantially higher than is observed in the absence of linkage. When one of the targets is an unlinked gene of invariant copy number, then the ratio (corrected for diploidy) between the gene of interest (for example, a KIR gene) and the invariant gene is a direct measure of the gene of interest copy number. In this proof of principle study, we show that ddPCR can be used to perform molecular haplotype analysis and CNV enumeration in the KIR system.

## Methods

### Droplet digital PCR

ddPCR was carried out using the QX100 Droplet Digital PCR system (Bio-Rad Laboratories, Hemel Hempstead, UK). ddPCR reactions were 22 μl aqueous volumes that contained final concentrations of 1X ddPCR supermix (Bio-Rad), 0.3 μM each primer and probe and 10 to 30 ng of genomic DNA. Droplet generation and droplet reading for ddPCR were carried out according to the manufacturer’s instructions using Bio-Rad reagents. The thermal cycling profile was 95°C:10′00′′ followed by 43 cycles of (95°C:0′15′′|50°C:1′00′′).

### *KIR2DL5* linkage analysis

Ten specimens of DNA derived from International Histocompatibility Workshop (IHW) cell lines were selected because standard quantitative PCR (qPCR) had indicated that they possessed at least one copy of *KIR2DL5* and either or both of *KIR2DL2* and *KIR3DS1*. DNA was extracted using the QIAamp DNA Blood Midi/Maxi kit (QIAGEN, Manchester, UK), which prepares DNA fragments up to 50 kb in length (QIAamp Blood Midi/Maxi kit handbook, 04/2010, QIAGEN). The presence of fragments longer than 23 kb was confirmed by gel electrophoresis and direct comparison to λHindIII/φX174HaeIII molecular weight markers. Two separate ddPCR assays were performed. Each of these was designed to detect one of the haplotypes *KIR2DL2* ~ *KIR2DL5C* (approximately 21.8 kb) or *KIR3DS1* ~ *KIR2DL5T (*approximately 19.9 kb)*.* Sequences of the KIR-specific oligonucleotides (Table S1 in Additional file 1) are identical to those used previously [10]. PCR probes were labeled with either 6-carboxyfluorescein (FAM; excitation 492 nm, emission 517 nm) or 6-carboxy-2,4,4,5,7,7-hexachlorofluorescein succinimidyl ester (HEX; excitation 535 nm, emission 553 nm). The probes hybridized to *KIR2DL2* exon 4, *KIR3DS1* exon 4 and *KIR2DL5* exon 9. Specimens were tested both before and after being subjected to restriction endonuclease digestion with 5U EcoRI (New England Biosciences, Hitchin, UK).

ddPCR data were collected and analyzed with QuantaLife® software V2.0 (BioRad Laboratories). Crosshair gating was used to split the data into four quadrants in a procedure analogous to that applied widely in flow cytometry analysis. Double negative (FAM^-^HEX^-^) droplets contained neither KIR gene target. HEX-positive droplets (FAM^-^HEX^+^) contained only *KIR2DL5*. FAM-positive droplets (FAM^+^HEX^-^) contained either *KIR2DL2* or *KIR3DS1* (depending on the assay) and double positive (FAM^+^HEX^+^) droplets contained both *KIR2DL5* and either of *KIR2DL2* or *KIR3DS1*. The primary measure of linkage between the two genes in the assay is the 'linkage' score, which is automatically calculated by the QuantaLife® software. The linkage score reports the estimated total number of molecules (copies/μl) in the assay that carry both target genes in linkage. Dividing this number by the total concentration of *KIR3DS1* or *KIR2DL2* determines the percentage of those genes in the test aliquot that are physically linked to *KIR2DL5*. This normalizes the linkage score for differences in the input amount of DNA between assays and we refer herein to %L as the normalized linkage score. After restriction endonuclease digestion, %L should be significantly decreased.

### *KIR2DL5* CNV analysis

Seven genomic DNA samples came from the UCLA international KIR exchange programme [37] and these were selected because they were recently extracted, high molecular weight DNA preparations from bulk cultures. Aliquots of the KIR exchange DNAs are also widely available in the KIR research community. Twenty-seven DNA samples were archival specimens that came from five Gambian nuclear families that were collected as part of another study (Medical Research Council Unit, The Gambia, study number SCC1177v2). The Ethics Committee of the Gambian Government/Medical Research Council Unit, and the ethics committee of the London School of Hygiene and Tropical Medicine approved the collection and genotyping of the Gambian samples. Individual written informed consent was obtained from all adult participants. Consent was obtained from a parent/guardian on behalf of those subjects aged <18 years who gave assent. All samples were anonymized. The Gambian specimens were collected in accordance with the tenets of the declaration of Helsinki.

An internal reference gene, the invariant single copy per haplotype ribonuclease P/MRP 30 kDa subunit (*RPP30*) gene, was assayed on the HEX channel as previously described [38]. Sequences of the KIR-specific oligonucleotides are identical to those used previously [10] (Additional file 1). *KIR2DL5* was assayed on the FAM channel. Counts of KIR-positive droplets (FAM^+^HEX^-^ and FAM^+^HEX^+^) and of *RPP30*-positive droplets (FAM^-^HEX^+^ and FAM^+^HEX^+^) were used to estimate the number of copies per microliter of each target in the reaction [35]. Estimates of the KIR target were divided by the estimates of the *RPP30* target, then multiplied by two to correct for diploidy in KIR CNV estimates. Clustering of raw CNV data into bins representing zero, one, two, three or four copies was performed by application of mclust, which permits a Gaussian finite mixture modeling via the expectation-maximization algorithm [39]. *KIR2DS3* and *KIR2DS5* genotyping was performed using a validated PCR, using sequence-specific primers (PCR-SSP) approach [40]. CNV variation in eight KIR genes (*KIR2DL1*, *KIR3DL1*, *KIR2DS1*, *KIR2DS2*, *KIR2DS3*, *KIR2DS5*, *KIR3DS1* and *KIR3DP1*; Figures S1 to S8 in Additional file 1) was tested using the same method for 19 samples from a number of sources. There were insufficient data to apply mclust to these data, so clustering into bins was performed by eye with the aid of the figures. qPCR and ddPCR results were compared using Cohen’s Kappa statistic.

## Results

### Genetic linkage analysis

**Figure 2:**
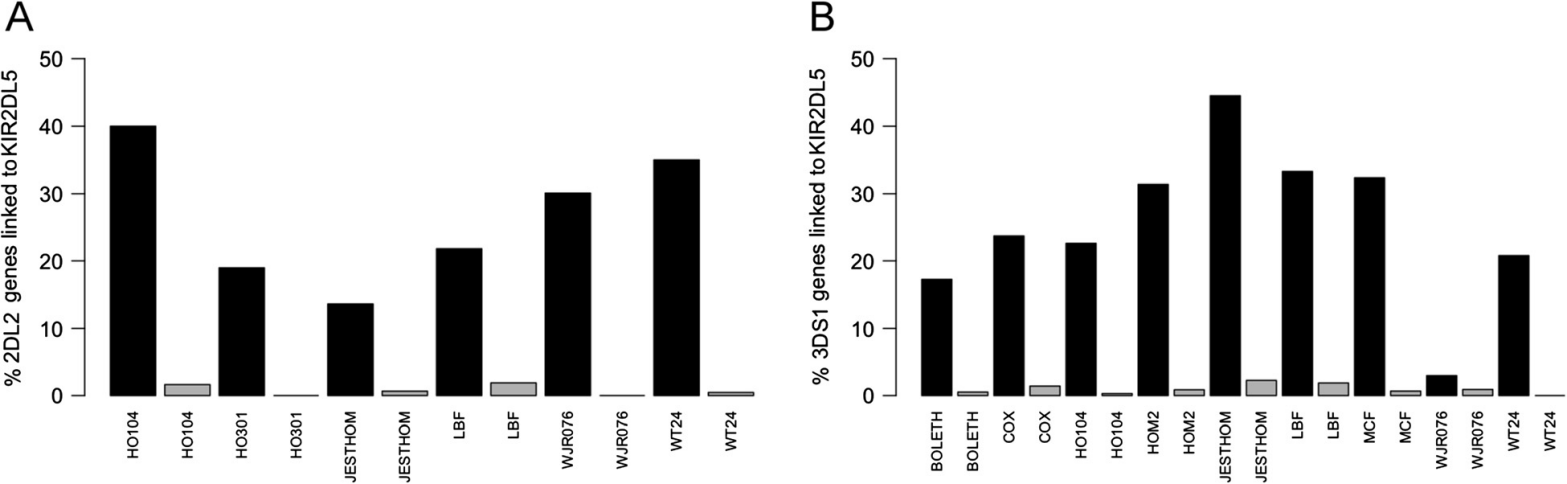
**Experimentally determined signatures of linkage between (A) *KIR2DL2 *and *KIR2DL5 *and (B) *KIR3DS1 *and *KIR2DL5 *for the 10 IHW cell lines.** Evidence of linkage (black bars) in DNA sample preparations (fragment size >23 kb), expressed as the percentage of *KIR2DL2* or *KIR3DS1* genes that were linked to a *KIR2DL5* gene. Linkage was abrogated by EcoRI restriction endonuclease digestion (gray bars).

The experimentally determined evidence for the presence or absence of each haplotype motif is shown in Figure 2. There was evidence for the presence of *KIR2DL2* ~ *KIR2DL5C* arrangement in the undigested DNA preparations of the cell lines HO104, HO301, JESTHOM, LBF (aka LBUF), WJR076 and WT24, whilst the remaining cell lines did not possess any copies of *KIR2DL2*. The *KIR3DS1* ~ *KIR2DL5T* haplotype was present in BOLETH, COX, HO104, HOM2, JESTHOM, LBF, MCF and WT24. HO301 did not have *KIR3DS1*. Evidence for linkage was partially or totally abrogated in the EcoRI digested DNA preparations.

### WJR076

In the case of WJR076, there was definitive evidence for the presence of the *KIR2DL2* ~ *KIR2DL5* haplotype (%L = 30, falling to %L = 0% after EcoRI digestion), which demonstrates that sample quality was sufficient to detect linkage over ~22 kb. In another aliquot from the same DNA source, there was some evidence for the presence of *KIR3DS1 ~ KIR2DL5* (%L = 2.9, falling to %L = 0.9% after EcoRI digestion). This may indicate linkage across a much greater distance.

### Copy number variation

**Figure 3:**
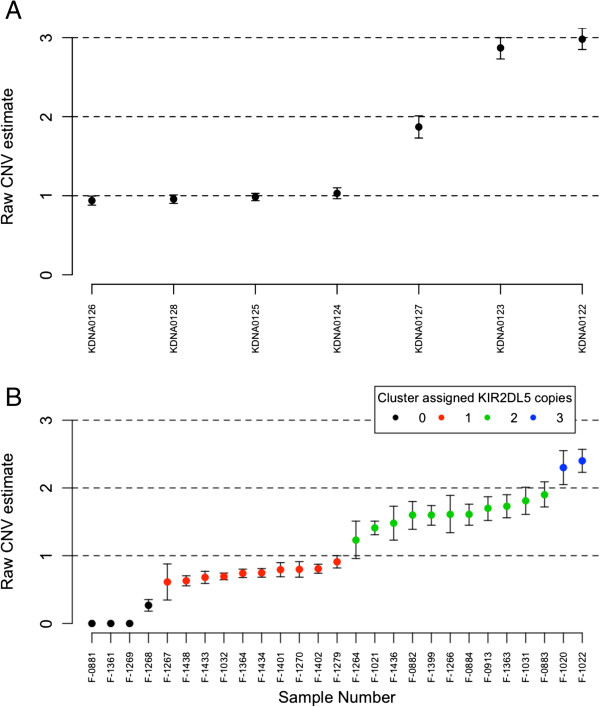
**KIR CNV estimates in (A) UCLA International KIR exchange samples and (B) Gambian family samples.** Colour-coded points indicate cluster membership (0 copies (n = 3), 1 copy (n = 10), 2 copies (n = 12), 3 copies (n = 2)).

**Figure 4:**
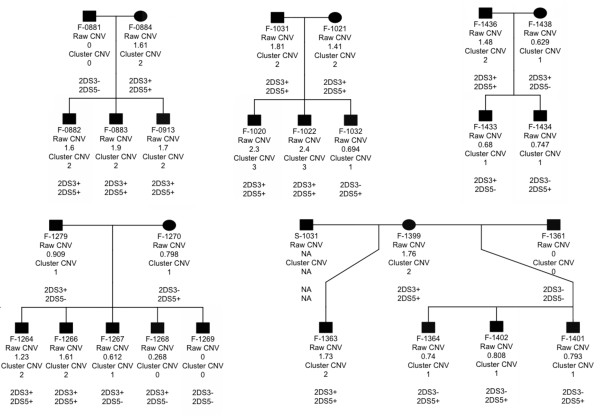
**Segregation of *KIR2DL5 *in five Gambian families.** Patterns of segregation are consistent with Mendelian inheritance and with segregation of the linked *KIR2DS3* and *KIR2DS5* genes. In most world populations, *KIR2DL5* is in perfect linkage disequilibrium with KIR2DS3 and *KIR2DS5*. In Gambia, these genes are in almost perfect linkage disequilibrium (Roberts C.h., Molina, S., Makalo, P., Joof, H., Harding-Esch, E.M., Burr, S.E., Mabey, D.W., Bailey, R.L., Burton, M.J. and Holland, M.J., in press). The absence of both *KIR2DS3* and *KIR2DS5* implies that *KIR2DL5* will be absent. The presence of both genes indicates that at least two *KIR2DL5* copies are present. The co-segregation of *KIR2DL5* with the *KIR2DS3* and *KIR2DS5* genes is expected and was observed in these pedigrees. To protect the anonymity of participant families, all F_1_ subjects have been changed to ‘male’.

Based on the ddPCR analysis, *KIR2DL5* was present at between zero and three copies in the specimens tested. The raw CNV estimates of the UCLA KIR exchange samples fit closely to integers (Figure 3A), whilst systematic underestimates of copy number were observed in samples from the Gambian families (Figure 3B). The colours of the points in Figure 3B reflect the results of applying mclust to the *KIR2DL5* CNV data. *KIR2DL5* CNVs segregated through the Gambian families in patterns that were compatible with Mendelian inheritance. *KIR2DL5* co-segregated with *KIR2DS3* and *KIR2DS5* (Figure 4), which are usually found in tight linkage disequilibrium with *KIR2DL5*[20]. One sample (F-1268) was assigned to the zero copy cluster by the mclust approach, but had a non-zero (0.268, confidence interval (CI) 0.182 to 0.353) CNV estimate, suggesting a true copy number of one. The CNV testing of eight additional KIR genes is shown in Figures S1 to S8 and Table S2 in Additional file 1).

## Discussion

Recent advances in KIR genotyping [10,17] have confirmed that CNVs are common in KIR genes and may relate directly to NK cell repertoire [41] and surface expression levels of the KIR proteins [42] with potential impact on NK cell function. We developed a next generation digital PCR assay for enumeration of KIR CNVs and resolution of KIR haplotype structures by genetic linkage analysis.

We have shown how ddPCR can be used to detect the presence or absence of the *KIR3DS1* ~ *KIR2DL5* (spanning approximately 21.8 kb) and *KIR2DL2* ~ *KIR2DL5* (spanning approximately 19.9 kb) groupings (Figure 2). Evidence for linkage was found in our ability to enumerate the percentage of *KIR2DL5* genes that were linked to another assayed gene. The application of EcoRI restriction endonuclease digestion abrogated these signatures of linkage and demonstrated the robustness of the method.

Proximity between the assayed genes, the variable copy number of each target and the average length of the DNA fragment in the tested material are all able to influence the %L score and they are likely to do so in a complex and unpredictable manner. When the distance between two targets is very high, the potential for mechanical shearing is increased and high %L scores are less likely. Low sample quality will reduce the maximum achievable %L score, whilst gene copy numbers will impose absolute limits in all specimens. For instance, a specimen with two copies of KIR2DL5 (one on each chromosome), but a single copy of KIR2DL2, will have a maximum possible %L of 50.

**Figure 5:**
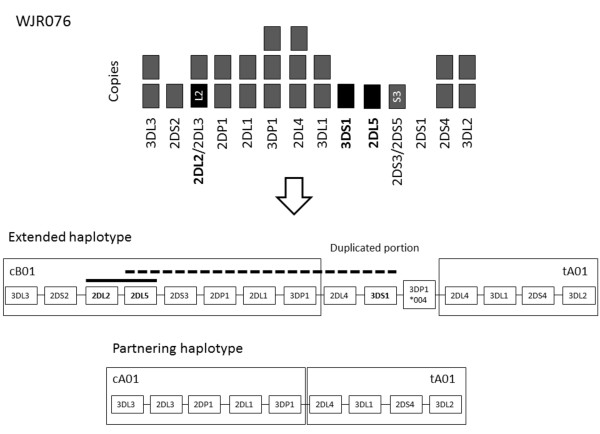
**The predicted gene carriage and resolved arrangements for WJR076 haplotypes.** Showing the constituent haplotype motifs: cB01:tA01 within the extended KIR B haplotype and cA01:tA01 forming a standard KIR A haplotype. The extended haplotype has the *2DL5/3DP1* hybrid gene (KIR3DP1*004) and duplication of the *KIR2DL4* and *KIR3DL1/S1* loci. Linkage analysis supported close relationship of *KIR2DL2* ~ *KIR2DL5* (indicated by a solid black line) and long-distance linkage between *KIR2DL5* and *KIR3DS1* (dashed line) on the extended haplotype.

In the case of one sample in our study, WJR076, we detected a weak %L score that was reduced by treatment with EcoRI. We suspected that this may be explained by the presence of an unusual extended haplotype and investigated this further. qPCR copy number data for WJR076 were consistent with the carriage of an extended KIR haplotype carrying a duplication of the *KIR2DL4* and *KIR3DL1/S1* loci. We subsequently ascertained by PCR-SSP that WJR076 carries the hybrid gene *KIR2DL5/3DP1* (known as KIR3DP1*004), which has been previously identified on various extended KIR haplotypes [9,10,43]. Our *KIR2DL5* ddPCR assay targets *KIR2DL5* exon 9, which is absent from KIR3DP1*004, so would not detect the portion of *KIR2DL5* (promoter region to exon 2) that forms part of the hybrid gene. The extended haplotype is, therefore, predicted to take the form *KIR2DL2* ~ *KIR2DL5* ~ […] ~ *KIR3DS1*, where the *KIR2DL2* ~ *KIR2DL5* conformation is in the centromeric motif and the *KIR3DS1* gene is in the extended region (Figure 5). This example illustrates how in future this approach will be useful for identifying and characterizing specimens, which may have novel haplotype structures. With improvements in DNA extraction procedures to yield longer unbroken fragments (>100 kb) the ddPCR technique will be able to link genes across the entire length of KIR haplotypes; however, we might expect very low %L scores in these samples, even when great care is taken during DNA preparation. The longest fragments will always be in the minority and there is likely to be a maximum feasible length of DNA fragment that can survive the mechanical forces involved in generating and manipulating the ddPCR droplets.

Pedigree analysis confirmed that ddPCR was an effective way to carry out CNV enumeration of *KIR2DL5*. We tested DNA samples of different origins, which had been extracted using different methods and some of which (the Gambian specimens) had been subjected to multiple freeze/thaw cycles and stored for prolonged periods of time. We found that there was variation in how well the estimated CNV values fit to integers (Figure 3) and that this was correlated with the source of the DNA (Figure 3A,B) as well as being influenced by the primer sets (Figures S4 and S7 in Additional file 1) and by sample to sample variation (Figures S1 to S8 in Additional file 1; note that specimen 10865 diverges from the integers in several assays, but 10834 fits closely to integers). We subjected aliquots of the UCLA specimens to 15 freeze-thaw cycles, but were unable to affect changes in the measured CNV estimates (data not shown). There were no systematic differences in the input amounts of DNA, although the measured concentrations of *RPP30* were systematically lower in the Gambian samples. This probably reflects that they came from buccal swab specimens and that a significant proportion of the total DNA is likely to be of bacterial origin. The CEPH and local donor DNA specimens (QPQ, NNA, CFF, AHS, JKN, RTC) had no systematic concentration differences from the UCLA samples, but still failed to fit to integers. The common features between the Gambian and CEPH/local donor specimens were their age and that they had been previously archived at -20°C so we might suspect that acid hydrolysis may be a factor for consideration. Further and careful investigation will be needed to identify the reasons for the variation in fit to integers, but a simple clustering approach was still able to discern the groups of samples with zero, one, two and three copies of *KIR2DL5*.

Assay-specific optimization will be necessary in order to fully establish ddPCR-based KIR CNV tests as routine tools, but the ddPCR CNV data compared well to a conventional qPCR method. Six assays had perfect agreement (Cohen’s Kappa = 1) between the two methods (*KIR2DL1*, *KIR2DS1*, KIR2DS2, *KIR2DS3*, *KIR2DS5*, *KIR3DS1*), whilst there were disagreements between the results of two assays, *KIR3DL1* (Kappa = 0.9, 95% CI 0.71 to 1.00) and *KIR3DP1* (Kappa = 0.74, 95% CI 0.49 to 0.99).

## Conclusions

The definition and characterization of structural haplotypes in the KIR region provide a significant challenge and one that has not previously been addressed using a simple, scalable, cost-effective and reproducible method. The next generation PCR approach described here makes it possible for the first time to routinely define KIR structural haplotypes in a way that is not possible using other PCR methods. In addition we have shown that ddPCR has potential for development as a method for KIR CNV enumeration.

We have shown that both the clustering method (Figures 3 and 4) and the use of raw data (Figure 4, kappa analysis) can achieve accurate CNV estimates. Clustering should be generally favored over the more subjective approach, but the example of the F-1268 specimen, which mclust very obviously placed in the wrong cluster, indicates that there will continue to be a role for human inspection in the classification process.

For several of the assays that we describe (Figures S1 to S3 and S6 in Additional file 1), the interpretation of ddPCR KIR CNV data was made easier by the simultaneous testing of a plurality of specimens. This is because sufficient data were generated to identify discrete clusters of data points (either by eye or using statistical clustering methods) even though the raw CNV estimates did not reproducibly fit to integers. In a clinical setting it is unlikely that large numbers of specimens will always need to be tested simultaneously and the variation between DNA from different sources may cause additional problems. A simple solution might be to utilize a supervised learning method wherein a large set of specimens are initially tested *in situ* at the clinic or centre, in order to train the algorithm for classification in a way that works given the operating procedure and in the context of the specific primer/probe set and how, on average, it performs given the average sample that will be tested at that specific centre. In application, it is also recommended that a set of control samples of known copy number are included in each run to be used as calibrators in the analysis and external quality control.

The gene linkage application will complement KIR genotype data and aid understanding of the population genetic and structural diversity of the KIR gene region. An immediate utility is that it allows KIR 'AB' and 'BB' genotypes to be distinguished, which are currently grouped together as 'Bx' because of uncertainty in KIR copy numbers. The KIR 'Bx' genotype has previously been associated with increased relapse-free survival after unrelated haematopoietic cell transplant [44] and the discrimination of 'AB' from 'BB' genotypes will make it possible to perform a more refined investigation of this association, including the determination of the underlying genetic model. The KIR 'AB' genotype has also been shown to predict higher *in vitro* cytokine responses to pathogen-associated signals in human KIR^+^ CD56^dim^ NK cells [45]. The assay described here will make it possible to specifically identify 'AB' individuals and to undertake genetic association studies that model this behaviour. Histocompatibility matching algorithms for haematopoietic stem cell transplants already take account of KIR genotypes [44] and the ability to perform assays such as those we describe on a high throughput platform will facilitate the routine consideration of KIR CNV diversity and haplotype diversity in the selection of appropriate donors. The affordability (less than $5 US per test) and scalability (one operator can run four 96-well plates in a normal working day) of the ddPCR assay makes it an excellent candidate platform for high throughput KIR CNV genotyping/haplotyping for transplantation registries and for use in large-scale genetic association and population genetics studies.

## Abbreviations

%L: percent linkage, the normalized linkage score; CI: confidence interval; CNV: copy number variation; ddPCR: droplet digital PCR; FAM: 6-carboxyfluorescein; HEX: 6-carboxy-2,4,4,5,7,7-hexachlorofluorescein succinimidyl ester; IHW: International Histocompatibility Workshop; KIR: Killer-cell Immunoglobulin-like Receptor; NK: natural killer; PCR: polymerase chain reaction; PCR-SSP: PCR, using sequence specific primers; qPCR: quantitative PCR.

## Competing interests

The authors declare that they have no competing interests.

## Authors’ contributions

ChR, JAT and WJ conceived the study. ChR, JAT, WJ and JJ performed the experiments. ChR and JAT analyzed the data. JT and MJH contributed reagents and samples. ChR, JAT, JT and MJH wrote the manuscript. All authors read, revised and approved the manuscript.

## Supplementary Material

Additional file 1: Table S1 Oligonucleotide primers and probes used in this study. **Table S2**. Summary of KIR CNV genotypes in 19 samples, tested using qPCR and ddPCR. **Table S3**. Droplet counts and lambda statistics for all tests carried out in this study. **Figure S1**. Results of ddPCR KIR CNV assay: *KIR2DL1*. **Figure S2**. Results of ddPCR KIR CNV assay: *KIR2DS1*. **Figure S3**. Results of ddPCR KIR CNV assay: *KIR2DS2*. **Figure S4**. Results of ddPCR KIR CNV assay: *KIR2DS3*. **Figure S5**. Results of ddPCR KIR CNV assay: *KIR2DS5*. **Figure S6**. Results of ddPCR KIR CNV assay: *KIR3DL1*. **Figure S7**. Results of ddPCR KIR CNV assay: *KIR3DP1*. **Figure S8**. Results of ddPCR KIR CNV assay: *KIR3DS1*. Click here for file
