# Supplementary material for: Killer-cell Immunoglobulin-like Receptor gene linkage and copy number variation analysis by droplet digital PCR
Source: Genome Med. 2014 Mar 5;6(3):20. doi: 10.1186/gm537 (PMC4062048; doi:10.1186/gm537)
Supplement: Additional file 1: Table S1 — Oligonucleotide primers and probes used in this study. Table S2. Summary of KIR CNV genotypes in 19 samples, tested using qPCR and ddPCR. Table S3. Droplet counts and lambda statistics for all tests carried out in this study. Figure S1. Results of ddPCR KIR CNV assay: KIR2DL1. Figure S2. Results of ddPCR KIR CNV assay: KIR2DS1. Figure S3. Results of ddPCR KIR CNV assay: KIR2DS2. Figure S4. Results of ddPCR KIR CNV assay: KIR2DS3. Figure S5. Results of ddPCR KIR CNV assay: KIR2DS5. Figure S6. Results of ddPCR KIR CNV assay: KIR3DL1. Figure S7. Results of ddPCR KIR CNV assay: KIR3DP1. Figure S8. Results of ddPCR KIR CNV assay: KIR3DS1. [file gm537-S1.docx]

**Additional Data : CNV analysis in other KIR genes.**

**Killer-cell Immunoglobulin-like Receptor gene linkage and copy number variation analysis by droplet digital PCR.**

Chrissy h. Roberts^1#^, Wei Jiang^2^, Jyothi Jayaraman^2^, John Trowsdale^2^, Martin J. Holland^1^ and James A. Traherne^2^

1. London School of Hygiene and Tropical Medicine, London, UK

2. Cambridge Institute for Medical Research, Addenbrooke’s Hospital, Cambridge, UK

Table S1. Oligonucleotide primers and probes for KIR are the same as used in Jiang et al. 2012

| Gene | Oligo-nucleotides | Sequence (5'-3') |
| --- | --- | --- |
| *KIR2DL5* | Primer 1 | CACTGCGTTTTCACACAGAC |
|  | Primer 2 | GGCAGGAGACAATGATCTT |
|  | Probe | CCCTTCTCAGAGGCCCAAGACACC |
| *KIR2DS2* | Primer 1 | GTCCCCTGGTGAAATCAGA |
|  | Primer 2 | TGAGGTGCAAAGTGTCCTTAT |
|  | Probe | TCATCCTGCAATGTTGGTCAGATGTCA |
| *KIR2DL1* | Primer 1 | TTCTCCATCAGTCGCATGAC |
|  | Primer 2 | GTCACTGGGAGCTGACAC |
|  | Probe | AACAGAACCGTAGCATCTGTAGGTCCCT |
| *KIR3DP1* | Primer 1 | GTCCCCTGGTGAAATCAGA |
|  | Primer 2 | GTGAGGCGCAAAGTGTCA |
|  | Probe | TCATCCTGCAATGTTGGTCAGATGTCA |
| *KIR3DL1* | Primer 1 | CATCGGTCCCATGATGCT |
|  | Primer 2 | GGGAGCTGACAACTGATAGG |
|  | Probe | AACAGAACCGTAGCATCTGTAGGTCCCT |
| *KIR3DS1* | Primer 1 | CATCGGTTCCATGATGCG |
|  | Primer 2 | GGGAGCTGACAACTGATAGG |
|  | Probe | AACAGAACCGTAGCATCTGTAGGTCCCT |
| *KIR2DS3* | Primer 1 | CTCCATCGGTCGCATGAG |
|  | Primer 2 | GGGTCACTGGGAGCTGAA |
|  | Probe | AACAGAACCGTAGCATCTGTAGGTCCCT |
| *KIR2DS5* | Primer 1 | AGAGAGGGGACGTTTAACC |
|  | Primer 2 | TCCAGAGGGTCACTGGGC |
|  | Probe | AACAGAACCGTAGCATCTGTAGGTCCCT |
| *KIR2DS1* | Primer 1 | TCTCCATCAGTCGCATGAA |
|  | Primer 2 | GGTCACTGGGAGCTGAC |
|  | Probe | AACAGAACCGTAGCATCTGTAGGTCCCT |
| *RPP30* | Primer 1 | AGATTTGGACCTGCGAGCG |
|  | Primer 2 | GAGCGGCTGTCTCCACAAGT |
|  | Probe | TTCTGACCTGAAGGCTCTGCGCG |

Table S2. Summary of KIR CNV genotypes in 19 samples, tested using qPCR and ddPCR.

|  | **2DL1** | | **2DS1** | | **2DS2** | | **2DS3** | | **2DS5** | | **3DL1** | | **3DP1** | | **3DS1** | |
| --- | --- | --- | --- | --- | --- | --- | --- | --- | --- | --- | --- | --- | --- | --- | --- | --- |
| **Specimen** | qPCR | ddPCR | qPCR | ddPCR | qPCR | ddPCR | qPCR | ddPCR | qPCR | ddPCR | qPCR | ddPCR | qPCR | ddPCR | qPCR | ddPCR |
| 07000 | 2 | 1.7 (1.57-1.85) | 1 | 0.83 (0.74-0.91) | 1 | 0.89 (0.81-0.98) | 0 | 0 (0-0) | 1 | 0.97 (0.88-1.08) | 1 | 0.85 (0.77-0.94) | 2 | 1.58 (1.46-1.72) | 1 | 0.94 (0.85-1.04) |
| 10834 | 2 | 1.85 (1.73-1.98) | 0 | 0 (0-0) | 1 | 0.89 (0.82-0.97) | 1 | 1 (0.92-1.08) | 0 | 0 (0-0) | 2 | 1.87 (1.75-2) | 3 | 1.98 (1.86-2.12) | 1 | 0.99 (0.91-1.08) |
| 10835 | 2 | 1.63 (1.52-1.73) | 1 | 0.69 (0.64-0.75) | 0 | 0 (0-0.01) | 0 | 0 (0-0) | 1 | 0.93 (0.86-1.01) | 1 | 0.82 (0.76-0.88) | 2 | 1.18 (1.1-1.26) | 1 | 0.96 (0.89-1.03) |
| 10858 | 2 | 1.71 (1.58-1.85) | 2 | 1.5 (1.38-1.63) | 1 | 0.82 (0.74-0.9) | 3 | 2.9 (2.68-3.13) | 0 | 0 (0-0) | 0 | 0 (0-0) | 2 | 1.22 (1.12-1.33) | 2 | 1.77 (1.62-1.92) |
| 10865 | 1 | 0.61 (0.57-0.65) | 1 | 0.4 (0.37-0.43) | 2 | 1.19 (1.12-1.25) | 1 | 0.9 (0.85-0.95) | 1 | 0.81 (0.76-0.87) | 1 | 0.49 (0.46-0.53) | 1 | 0.29 (0.27-0.31) | 0 | 0 (0-0) |
| 11879 | 2 | 1.87 (1.68-2.08) | 1 | 0.75 (0.66-0.85) | 0 | 0.01 (0-0.01) | 1 | 0.99 (0.87-1.12) | 0 | 0 (0-0) | 1 | 0.9 (0.8-1.01) | 2 | 1.31 (1.18-1.45) | 1 | 1.04 (0.92-1.18) |
| 11880 | 2 | 1.91 (1.76-2.07) | 1 | 0.78 (0.7-0.87) | 1 | 0.9 (0.83-0.98) | 2 | 1.93 (1.78-2.09) | 0 | 0 (0-0) | 1 | 0.97 (0.87-1.07) | 2 | 1.15 (1.07-1.23) | 1 | 1.07 (0.96-1.19) |
| 11881 | 1 | 0.79 (0.73-0.85) | 0 | 0 (0-0) | 1 | 0.85 (0.79-0.92) | 0 | 0 (0-0) | 0 | 0 (0-0) | 2 | 1.68 (1.58-1.79) | 2 | 1.04 (0.97-1.11) | 0 | 0 (0-0) |
| 11882 | 1 | 0.89 (0.8-0.98) | 0 | 0 (0-0) | 2 | 1.34 (1.19-1.5) | 0 | 0 (0-0) | 0 | 0 (0-0) | 3 | 2.25 (2.03-2.49) | 3 | 2.12 (1.92-2.33) | 0 | 0 (0-0) |
| 11891 | 1 | 0.96 (0.86-1.06) | 1 | 0.63 (0.57-0.68) | 1 | 0.85 (0.79-0.92) | 0 | 0 (0-0) | 1 | 1.01 (0.93-1.09) | 1 | 0.81 (0.75-0.87) | 1 | 0.54 (0.5-0.58) | 0 | 0 (0-0) |
| 11892 | 2 | 1.53 (1.44-1.63) | 0 | 0 (0-0) | 2 | 1.78 (1.66-1.9) | 2 | 1.95 (1.83-2.08) | 0 | 0 (0-0) | 2 | 1.53 (1.43-1.64) | 2 | 1.13 (1.05-1.2) | 0 | 0 (0-0) |
| 12109 | 1 | 0.97 (0.88-1.06) | 1 | 0.7 (0.63-0.77) | 0 | 0 (0-0.01) | 0 | 0 (0-0) | 0 | 0 (0-0) | 1 | 0.95 (0.86-1.04) | 1 | 0.59 (0.53-0.65) | 0 | 0 (0-0) |
| 12248 | 2 | 1.77 (1.65-1.89) | 1 | 0.53 (0.49-0.58) | 0 | 0 (0-0.01) | 0 | 0 (0-0) | 1 | 0.98 (0.91-1.05) | 1 | 0.69 (0.64-0.75) | 2 | 1 (0.93-1.07) | 1 | 0.66 (0.58-0.74) |
| QPQ | 2 | 1.95 (1.78-2.13) | 2 | 1.64 (1.5-1.8) | 1 | 0.88 (0.79-0.98) | 1 | 0.97 (0.87-1.07) | 2 | 1.98 (1.82-2.15) | 0 | 0 (0-0) | 3 | 2.1 (1.94-2.27) | 3 | 2.89 (2.69-3.11) |
| NNA | 0 | 0 (0-0.01) | 0 | 0 (0-0) | 2 | 2 (1.88-2.13) | 0 | 0 (0-0) | 0 | 0 (0-0) | 2 | 1.91 (1.78-2.04) | 2 | 1.5 (1.39-1.62) | 0 | 0 (0-0) |
| CFF | 1 | 0.91 (0.83-0.98) | 0 | 0 (0-0) | 2 | 1.92 (1.79-2.07) | 0 | 0 (0-0) | 0 | 0 (0-0) | 2 | 2.08 (1.91-2.27) | 2 | 1.65 (1.51-1.81) | 0 | 0 (0-0) |
| AHS | 2 | 1.92 (1.8-2.05) | 1 | 0.68 (0.62-0.74) | 1 | 0.91 (0.85-0.97) | 1 | 1.02 (0.95-1.1) | 1 | 0.99 (0.91-1.07) | 1 | 1 (0.92-1.09) | 3 | 1.69 (1.59-1.79) | 2 | 1.98 (1.85-2.12) |
| JKN | 2 | 1.6 (1.51-1.71) | 1 | 0.65 (0.6-0.7) | 0 | 0 (0-0.01) | 0 | 0 (0-0) | 1 | 0.94 (0.88-1.01) | 1 | 0.92 (0.86-0.99) | 2 | 1.28 (1.2-1.37) | 1 | 0.99 (0.93-1.07) |
| RTC | 2 | 2.03 (1.9-2.17) | 0 | 0 (0-0) | 0 | 0.01 (0-0.01) | 0 | 0 (0-0) | 0 | 0 (0-0) | 2 | 1.99 (1.87-2.12) | 2 | ND | 0 | 0 (0-0) |

**Table S3. Droplet counts and lambda statistics for all tests carried out in this study.**

Lambda is the mean number of copies of the targets per partition. Lambda values for FAM and HEX channels are reported here with the total droplet count for compliance with the suggested Digital MIQE standards (Huggett et al., 2013).

| SAMPLE | EcoRI | ASSAY | FAM+HEX+ | FAM+HEX- | FAM-HEX+ | FAM-HEX- | TOTAL | LAMBDA  FAM | LAMBDA  HEX |
| --- | --- | --- | --- | --- | --- | --- | --- | --- | --- |
| BOLETH | NO | 3DS1~2DL5 | 83 | 346 | 336 | 14390 | 15155 | 0.028 | 0.028 |
| BOLETH | YES | 3DS1~2DL5 | 11 | 329 | 411 | 14678 | 15429 | 0.022 | 0.027 |
| COX | NO | 3DS1~2DL5 | 118 | 346 | 315 | 16532 | 17311 | 0.027 | 0.025 |
| COX | YES | 3DS1~2DL5 | 9 | 272 | 274 | 14929 | 15484 | 0.018 | 0.018 |
| HO104 | NO | 3DS1~2DL5 | 227 | 539 | 1207 | 13065 | 15038 | 0.051 | 0.095 |
| HO104 | YES | 3DS1~2DL5 | 70 | 679 | 1426 | 14297 | 16472 | 0.045 | 0.091 |
| HOM2 | NO | 3DS1~2DL5 | 668 | 1114 | 1025 | 13125 | 15932 | 0.112 | 0.106 |
| HOM2 | YES | 3DS1~2DL5 | 149 | 1355 | 1319 | 13194 | 16017 | 0.094 | 0.092 |
| JESTHOM | NO | 3DS1~2DL5 | 855 | 797 | 1621 | 13072 | 16345 | 0.101 | 0.151 |
| JESTHOM | YES | 3DS1~2DL5 | 206 | 791 | 2741 | 11867 | 15605 | 0.064 | 0.189 |
| LBF | NO | 3DS1~2DL5 | 255 | 383 | 1493 | 14288 | 16419 | 0.039 | 0.106 |
| LBF | YES | 3DS1~2DL5 | 18 | 257 | 745 | 14942 | 15962 | 0.017 | 0.048 |
| MCF | NO | 3DS1~2DL5 | 249 | 471 | 427 | 15283 | 16430 | 0.044 | 0.041 |
| MCF | YES | 3DS1~2DL5 | 40 | 727 | 691 | 14463 | 15921 | 0.048 | 0.046 |
| WJR076 | NO | 3DS1~2DL5 | 53 | 609 | 680 | 12592 | 13934 | 0.048 | 0.053 |
| WJR076 | YES | 3DS1~2DL5 | 34 | 623 | 626 | 13975 | 15258 | 0.043 | 0.043 |
| WT24 | NO | 3DS1~2DL5 | 8 | 30 | 45 | 14138 | 14221 | 0.003 | 0.004 |
| WT24 | YES | 3DS1~2DL5 | 0 | 72 | 62 | 12499 | 12633 | 0.006 | 0.005 |
| HO104 | NO | 2DL2~2DL5 | 422 | 491 | 994 | 10154 | 12061 | 0.076 | 0.117 |
| HO104 | YES | 2DL2~2DL5 | 43 | 436 | 798 | 9937 | 11214 | 0.043 | 0.075 |
| HO301 | NO | 2DL2~2DL5 | 112 | 330 | 747 | 9267 | 10456 | 0.042 | 0.082 |
| HO301 | YES | 2DL2~2DL5 | 48 | 658 | 1387 | 13891 | 15984 | 0.044 | 0.09 |
| JESTHOM | NO | 2DL2~2DL5 | 222 | 1050 | 487 | 12083 | 13842 | 0.092 | 0.051 |
| JESTHOM | YES | 2DL2~2DL5 | 62 | 1085 | 626 | 12538 | 14311 | 0.08 | 0.048 |
| LBF | NO | 2DL2~2DL5 | 219 | 505 | 1422 | 12322 | 14468 | 0.05 | 0.113 |
| LBF | YES | 2DL2~2DL5 | 89 | 689 | 1423 | 13257 | 15458 | 0.05 | 0.098 |
| WJR076 | NO | 2DL2~2DL5 | 594 | 873 | 1864 | 11972 | 15303 | 0.096 | 0.161 |
| WJR076 | YES | 2DL2~2DL5 | 128 | 1067 | 1676 | 10936 | 13807 | 0.087 | 0.131 |
| WT24 | NO | 2DL2~2DL5 | 281 | 462 | 522 | 14069 | 15334 | 0.048 | 0.052 |
| WT24 | YES | 2DL2~2DL5 | 47 | 747 | 792 | 13677 | 15263 | 0.052 | 0.055 |
| 07000 | NO | 2DL1_CNV | 67 | 2004 | 2336 | 10907 | 15314 | 0.135 | 0.157 |
| 10834 | NO | 2DL1_CNV | 629 | 2667 | 2894 | 7756 | 13946 | 0.236 | 0.253 |
| 10835 | NO | 2DL1_CNV | 657 | 2830 | 3486 | 5616 | 12589 | 0.277 | 0.329 |
| 10858 | NO | 2DL1_CNV | 279 | 1990 | 2339 | 10974 | 15582 | 0.146 | 0.168 |
| 10865 | NO | 2DL1_CNV | 617 | 1820 | 5906 | 5474 | 13817 | 0.176 | 0.472 |
| 11879 | NO | 2DL1_CNV | 85 | 1245 | 1330 | 10184 | 12844 | 0.104 | 0.11 |
| 11880 | NO | 2DL1_CNV | 214 | 2093 | 2197 | 10464 | 14968 | 0.154 | 0.161 |
| 11881 | NO | 2DL1_CNV | 319 | 1543 | 3965 | 9736 | 15563 | 0.12 | 0.275 |
| 11882 | NO | 2DL1_CNV | 119 | 955 | 2187 | 10321 | 13582 | 0.079 | 0.17 |
| 11891 | NO | 2DL1_CNV | 463 | 593 | 1563 | 4608 | 7227 | 0.146 | 0.28 |
| 11892 | NO | 2DL1_CNV | 512 | 3039 | 3958 | 8594 | 16103 | 0.221 | 0.278 |
| 12109 | NO | 2DL1_CNV | 368 | 1027 | 2315 | 6658 | 10368 | 0.135 | 0.259 |
| 12248 | NO | 2DL1_CNV | 816 | 2454 | 2820 | 7258 | 13348 | 0.245 | 0.272 |
| QPQ | NO | 2DL1_CNV | 263 | 1650 | 1698 | 9741 | 13352 | 0.143 | 0.147 |
| NNA | NO | 2DL1_CNV | 0 | 10 | 3743 | 10951 | 14704 | 0.001 | 0.255 |
| CFF | NO | 2DL1_CNV | 287 | 1298 | 2984 | 9846 | 14415 | 0.11 | 0.227 |
| AHS | NO | 2DL1_CNV | 920 | 2609 | 2728 | 7407 | 13664 | 0.258 | 0.267 |
| JKN | NO | 2DL1_CNV | 670 | 2982 | 3720 | 6121 | 13493 | 0.271 | 0.325 |
| RTC | NO | 2DL1_CNV | 827 | 2819 | 2768 | 8385 | 14799 | 0.246 | 0.243 |
| 07000 | NO | 2DS1_CNV | 7 | 1015 | 2352 | 11868 | 15242 | 0.067 | 0.155 |
| 10834 | NO | 2DS1_CNV | 0 | 0 | 3537 | 11557 | 15094 | 0 | 0.234 |
| 10835 | NO | 2DS1_CNV | 28 | 1532 | 4055 | 9116 | 14731 | 0.106 | 0.277 |
| 10858 | NO | 2DS1_CNV | 10 | 1937 | 2526 | 10415 | 14888 | 0.131 | 0.17 |
| 10865 | NO | 2DS1_CNV | 15 | 1627 | 6574 | 6459 | 14675 | 0.112 | 0.449 |
| 11879 | NO | 2DS1_CNV | 0 | 597 | 1539 | 11698 | 13834 | 0.043 | 0.111 |
| 11880 | NO | 2DS1_CNV | 2 | 970 | 2351 | 10577 | 13900 | 0.07 | 0.169 |
| 11881 | NO | 2DS1_CNV | 0 | 0 | 4161 | 11304 | 15465 | 0 | 0.269 |
| 11882 | NO | 2DS1_CNV | 0 | 0 | 1822 | 13393 | 15215 | 0 | 0.12 |
| 11891 | NO | 2DS1_CNV | 42 | 1315 | 3849 | 8639 | 13845 | 0.098 | 0.281 |
| 11892 | NO | 2DS1_CNV | 0 | 0 | 3979 | 10770 | 14749 | 0 | 0.27 |
| 12109 | NO | 2DS1_CNV | 29 | 967 | 2558 | 6301 | 9855 | 0.101 | 0.263 |
| 12248 | NO | 2DS1_CNV | 28 | 1262 | 4224 | 8570 | 14084 | 0.092 | 0.302 |
| QPQ | NO | 2DS1_CNV | 29 | 1654 | 1989 | 8939 | 12611 | 0.133 | 0.16 |
| NNA | NO | 2DS1_CNV | 0 | 0 | 3524 | 9826 | 13350 | 0 | 0.264 |
| CFF | NO | 2DS1_CNV | 0 | 1 | 3198 | 10893 | 14092 | 0 | 0.227 |
| AHS | NO | 2DS1_CNV | 16 | 1293 | 3463 | 7837 | 12609 | 0.104 | 0.276 |
| JKN | NO | 2DS1_CNV | 28 | 1551 | 4236 | 6849 | 12664 | 0.125 | 0.337 |
| RTC | NO | 2DS1_CNV | 0 | 0 | 3201 | 10494 | 13695 | 0 | 0.234 |
| 07000 | NO | 2DS2_CNV | 87 | 1029 | 2303 | 11643 | 15062 | 0.074 | 0.159 |
| 10834 | NO | 2DS2_CNV | 282 | 1356 | 3124 | 9319 | 14081 | 0.116 | 0.242 |
| 10835 | NO | 2DS2_CNV | 0 | 9 | 4084 | 10863 | 14956 | 0.001 | 0.273 |
| 10858 | NO | 2DS2_CNV | 114 | 873 | 2188 | 11745 | 14920 | 0.066 | 0.154 |
| 10865 | NO | 2DS2_CNV | 1862 | 2729 | 4833 | 2786 | 12210 | 0.376 | 0.548 |
| 11879 | NO | 2DS2_CNV | 0 | 5 | 1805 | 12251 | 14061 | 0 | 0.128 |
| 11880 | NO | 2DS2_CNV | 272 | 1324 | 3006 | 8722 | 13324 | 0.12 | 0.246 |
| 11881 | NO | 2DS2_CNV | 374 | 1431 | 3498 | 8675 | 13978 | 0.129 | 0.277 |
| 11882 | NO | 2DS2_CNV | 66 | 857 | 1291 | 10810 | 13024 | 0.071 | 0.104 |
| 11891 | NO | 2DS2_CNV | 565 | 1512 | 3795 | 7350 | 13222 | 0.157 | 0.33 |
| 11892 | NO | 2DS2_CNV | 582 | 2855 | 3233 | 9228 | 15898 | 0.216 | 0.24 |
| 12109 | NO | 2DS2_CNV | 0 | 7 | 2760 | 6401 | 9168 | 0.001 | 0.301 |
| 12248 | NO | 2DS2_CNV | 0 | 14 | 4856 | 10634 | 15504 | 0.001 | 0.313 |
| QPQ | NO | 2DS2_CNV | 90 | 913 | 2074 | 9552 | 12629 | 0.079 | 0.171 |
| NNA | NO | 2DS2_CNV | 988 | 3050 | 3047 | 7261 | 14346 | 0.281 | 0.281 |
| CFF | NO | 2DS2_CNV | 482 | 2245 | 2340 | 8326 | 13393 | 0.204 | 0.211 |
| AHS | NO | 2DS2_CNV | 773 | 1820 | 4347 | 7666 | 14606 | 0.178 | 0.351 |
| JKN | NO | 2DS2_CNV | 1 | 10 | 4375 | 9290 | 13676 | 0.001 | 0.32 |
| RTC | NO | 2DS2_CNV | 0 | 19 | 3893 | 10615 | 14527 | 0.001 | 0.268 |
| 07000 | NO | 2DS3_CNV | 0 | 0 | 2291 | 13451 | 15742 | 0 | 0.146 |
| 10834 | NO | 2DS3_CNV | 346 | 1282 | 2740 | 10435 | 14803 | 0.11 | 0.208 |
| 10835 | NO | 2DS3_CNV | 0 | 0 | 4207 | 10247 | 14454 | 0 | 0.291 |
| 10858 | NO | 2DS3_CNV | 484 | 2552 | 1697 | 8409 | 13142 | 0.231 | 0.166 |
| 10865 | NO | 2DS3_CNV | 1469 | 1994 | 5129 | 5692 | 14284 | 0.242 | 0.462 |
| 11879 | NO | 2DS3_CNV | 76 | 616 | 1289 | 10875 | 12856 | 0.054 | 0.106 |
| 11880 | NO | 2DS3_CNV | 422 | 1908 | 1985 | 9590 | 13905 | 0.168 | 0.173 |
| 11881 | NO | 2DS3_CNV | 0 | 1 | 3804 | 11058 | 14863 | 0 | 0.256 |
| 11882 | NO | 2DS3_CNV | 0 | 0 | 1729 | 12499 | 14228 | 0 | 0.122 |
| 11891 | NO | 2DS3_CNV | 0 | 0 | 4019 | 9529 | 13548 | 0 | 0.297 |
| 11892 | NO | 2DS3_CNV | 797 | 2873 | 2948 | 8488 | 15106 | 0.243 | 0.248 |
| 12109 | NO | 2DS3_CNV | 0 | 0 | 2523 | 7267 | 9790 | 0 | 0.258 |
| 12248 | NO | 2DS3_CNV | 0 | 0 | 4093 | 9291 | 13384 | 0 | 0.306 |
| QPQ | NO | 2DS3_CNV | 162 | 894 | 1925 | 9524 | 12505 | 0.084 | 0.167 |
| NNA | NO | 2DS3_CNV | 0 | 0 | 4221 | 10461 | 14682 | 0 | 0.287 |
| CFF | NO | 2DS3_CNV | 0 | 0 | 3327 | 12338 | 15665 | 0 | 0.212 |
| AHS | NO | 2DS3_CNV | 693 | 1656 | 3527 | 7991 | 13867 | 0.169 | 0.304 |
| JKN | NO | 2DS3_CNV | 1 | 0 | 4540 | 8997 | 13538 | 0 | 0.335 |
| RTC | NO | 2DS3_CNV | 0 | 0 | 3739 | 10591 | 14330 | 0 | 0.261 |
| 07000 | NO | 2DS5_CNV | 119 | 924 | 1941 | 11137 | 14121 | 0.074 | 0.146 |
| 10834 | NO | 2DS5_CNV | 0 | 0 | 3174 | 9731 | 12905 | 0 | 0.246 |
| 10835 | NO | 2DS5_CNV | 320 | 1502 | 3284 | 8553 | 13659 | 0.133 | 0.264 |
| 10858 | NO | 2DS5_CNV | 0 | 0 | 2334 | 11673 | 14007 | 0 | 0.167 |
| 10865 | NO | 2DS5_CNV | 777 | 1936 | 4911 | 5452 | 13076 | 0.207 | 0.435 |
| 11879 | NO | 2DS5_CNV | 0 | 0 | 1488 | 12490 | 13978 | 0 | 0.106 |
| 11880 | NO | 2DS5_CNV | 0 | 0 | 2019 | 10732 | 12751 | 0 | 0.158 |
| 11881 | NO | 2DS5_CNV | 0 | 0 | 3971 | 11447 | 15418 | 0 | 0.258 |
| 11882 | NO | 2DS5_CNV | 0 | 0 | 1614 | 12136 | 13750 | 0 | 0.117 |
| 11891 | NO | 2DS5_CNV | 601 | 1458 | 3182 | 8355 | 13596 | 0.151 | 0.278 |
| 11892 | NO | 2DS5_CNV | 0 | 0 | 3972 | 11769 | 15741 | 0 | 0.252 |
| 12109 | NO | 2DS5_CNV | 0 | 0 | 2359 | 8003 | 10362 | 0 | 0.228 |
| 12248 | NO | 2DS5_CNV | 571 | 1542 | 3368 | 6833 | 12314 | 0.172 | 0.32 |
| QPQ | NO | 2DS5_CNV | 354 | 1879 | 1899 | 9624 | 13756 | 0.162 | 0.164 |
| NNA | NO | 2DS5_CNV | 1 | 0 | 3671 | 10840 | 14512 | 0 | 0.253 |
| CFF | NO | 2DS5_CNV | 1 | 0 | 2240 | 4586 | 6827 | 0 | 0.328 |
| AHS | NO | 2DS5_CNV | 453 | 1389 | 3018 | 8888 | 13748 | 0.134 | 0.252 |
| JKN | NO | 2DS5_CNV | 829 | 1574 | 3754 | 6927 | 13084 | 0.184 | 0.35 |
| RTC | NO | 2DS5_CNV | 0 | 0 | 3580 | 10388 | 13968 | 0 | 0.256 |
| 07000 | NO | 3DL1_CNV | 32 | 953 | 2180 | 12156 | 15321 | 0.064 | 0.144 |
| 10834 | NO | 3DL1_CNV | 680 | 2678 | 2875 | 9210 | 15443 | 0.217 | 0.23 |
| 10835 | NO | 3DL1_CNV | 378 | 1531 | 3872 | 9398 | 15179 | 0.126 | 0.28 |
| 10858 | NO | 3DL1_CNV | 0 | 0 | 2344 | 13308 | 15652 | 0 | 0.15 |
| 10865 | NO | 3DL1_CNV | 1001 | 1526 | 6584 | 3894 | 13005 | 0.194 | 0.583 |
| 11879 | NO | 3DL1_CNV | 40 | 714 | 1585 | 12043 | 14382 | 0.052 | 0.113 |
| 11880 | NO | 3DL1_CNV | 108 | 1004 | 2100 | 12052 | 15264 | 0.073 | 0.145 |
| 11881 | NO | 3DL1_CNV | 457 | 2985 | 3540 | 8058 | 15040 | 0.229 | 0.266 |
| 11882 | NO | 3DL1_CNV | 90 | 1535 | 1365 | 11465 | 14455 | 0.112 | 0.101 |
| 11891 | NO | 3DL1_CNV | 715 | 1386 | 3901 | 7564 | 13566 | 0.155 | 0.34 |
| 11892 | NO | 3DL1_CNV | 244 | 2648 | 3420 | 9205 | 15517 | 0.186 | 0.236 |
| 12109 | NO | 3DL1_CNV | 317 | 1036 | 2325 | 6190 | 9868 | 0.137 | 0.268 |
| 12248 | NO | 3DL1_CNV | 241 | 1301 | 3754 | 8533 | 13829 | 0.112 | 0.289 |
| QPQ | NO | 3DL1_CNV | 0 | 1 | 2632 | 11735 | 14368 | 0 | 0.183 |
| NNA | NO | 3DL1_CNV | 857 | 2493 | 2636 | 7525 | 13511 | 0.248 | 0.259 |
| CFF | NO | 3DL1_CNV | 308 | 1800 | 1723 | 8814 | 12645 | 0.167 | 0.161 |
| AHS | NO | 3DL1_CNV | 394 | 1306 | 2764 | 7790 | 12254 | 0.139 | 0.258 |
| JKN | NO | 3DL1_CNV | 696 | 1680 | 3964 | 8092 | 14432 | 0.165 | 0.323 |
| RTC | NO | 3DL1_CNV | 914 | 2743 | 2758 | 7735 | 14150 | 0.258 | 0.26 |
| 07000 | NO | 3DP1_CNV | 11 | 2147 | 2665 | 11208 | 16031 | 0.135 | 0.167 |
| 10834 | NO | 3DP1_CNV | 14 | 3611 | 3635 | 7562 | 14822 | 0.245 | 0.246 |
| 10835 | NO | 3DP1_CNV | 44 | 2776 | 4435 | 8752 | 16007 | 0.176 | 0.28 |
| 10858 | NO | 3DP1_CNV | 10 | 1674 | 2651 | 10207 | 14542 | 0.116 | 0.183 |
| 10865 | NO | 3DP1_CNV | 8 | 1319 | 6855 | 4865 | 13047 | 0.102 | 0.526 |
| 11879 | NO | 3DP1_CNV | 0 | 1226 | 1829 | 11770 | 14825 | 0.083 | 0.123 |
| 11880 | NO | 3DP1_CNV | 7 | 2369 | 3863 | 7531 | 13770 | 0.173 | 0.281 |
| 11881 | NO | 3DP1_CNV | 29 | 2561 | 4549 | 7919 | 15058 | 0.172 | 0.304 |
| 11882 | NO | 3DP1_CNV | 14 | 1674 | 1587 | 9387 | 12662 | 0.133 | 0.126 |
| 11891 | NO | 3DP1_CNV | 51 | 1401 | 4633 | 8231 | 14316 | 0.101 | 0.327 |
| 11892 | NO | 3DP1_CNV | 20 | 2644 | 4386 | 8101 | 15151 | 0.176 | 0.291 |
| 12109 | NO | 3DP1_CNV | 32 | 1020 | 3081 | 5116 | 9249 | 0.114 | 0.337 |
| 12248 | NO | 3DP1_CNV | 39 | 2473 | 4571 | 8314 | 15397 | 0.163 | 0.299 |
| QPQ | NO | 3DP1_CNV | 24 | 2559 | 2451 | 6863 | 11897 | 0.217 | 0.208 |
| NNA | NO | 3DP1_CNV | 24 | 2409 | 3112 | 7777 | 13322 | 0.183 | 0.235 |
| CFF | NO | 3DP1_CNV | 3 | 1739 | 2075 | 9042 | 12859 | 0.135 | 0.162 |
| AHS | NO | 3DP1_CNV | 101 | 4393 | 5061 | 5076 | 14631 | 0.307 | 0.353 |
| JKN | NO | 3DP1_CNV | 35 | 3107 | 4543 | 6340 | 14025 | 0.224 | 0.326 |
| RTC | NO | 3DP1_CNV | 0 | 4288 | 0 | 10498 | 14786 | 0.29 | 0 |
| 07000 | NO | 3DS1_CNV | 136 | 957 | 2088 | 11864 | 15045 | 0.073 | 0.148 |
| 10834 | NO | 3DS1_CNV | 337 | 1285 | 2731 | 8952 | 13305 | 0.122 | 0.231 |
| 10835 | NO | 3DS1_CNV | 575 | 1661 | 3737 | 10013 | 15986 | 0.14 | 0.27 |
| 10858 | NO | 3DS1_CNV | 331 | 1744 | 1994 | 10139 | 14208 | 0.146 | 0.164 |
| 10865 | NO | 3DS1_CNV | 0 | 0 | 8072 | 5041 | 13113 | 0 | 0.616 |
| 11879 | NO | 3DS1_CNV | 64 | 681 | 1331 | 12278 | 14354 | 0.052 | 0.097 |
| 11880 | NO | 3DS1_CNV | 119 | 934 | 1786 | 12167 | 15006 | 0.07 | 0.127 |
| 11881 | NO | 3DS1_CNV | 0 | 0 | 4707 | 10731 | 15438 | 0 | 0.305 |
| 11882 | NO | 3DS1_CNV | 0 | 0 | 1439 | 12477 | 13916 | 0 | 0.103 |
| 11891 | NO | 3DS1_CNV | 0 | 0 | 4151 | 11070 | 15221 | 0 | 0.273 |
| 11892 | NO | 3DS1_CNV | 0 | 0 | 4251 | 11934 | 16185 | 0 | 0.263 |
| 12109 | NO | 3DS1_CNV | 0 | 0 | 2712 | 7241 | 9953 | 0 | 0.272 |
| 12248 | NO | 3DS1_CNV | 58 | 587 | 1780 | 7345 | 9770 | 0.066 | 0.188 |
| QPQ | NO | 3DS1_CNV | 583 | 2886 | 1918 | 8834 | 14221 | 0.244 | 0.176 |
| NNA | NO | 3DS1_CNV | 0 | 0 | 4008 | 10999 | 15007 | 0 | 0.267 |
| CFF | NO | 3DS1_CNV | 0 | 1 | 2678 | 11871 | 14550 | 0 | 0.184 |
| AHS | NO | 3DS1_CNV | 672 | 2587 | 2611 | 8905 | 14775 | 0.221 | 0.222 |
| JKN | NO | 3DS1_CNV | 810 | 1548 | 3495 | 6991 | 12844 | 0.184 | 0.335 |
| RTC | NO | 3DS1_CNV | 0 | 1 | 3666 | 10457 | 14124 | 0 | 0.26 |
| F-0882 | NO | 2DL5_CNV | 8 | 423 | 530 | 13736 | 14697 | 0.029 | 0.037 |
| F-0883 | NO | 2DL5_CNV | 41 | 767 | 807 | 12537 | 14152 | 0.057 | 0.06 |
| F-0884 | NO | 2DL5_CNV | 55 | 710 | 890 | 12530 | 14185 | 0.054 | 0.067 |
| F-0913 | NO | 2DL5_CNV | 30 | 625 | 739 | 15083 | 16477 | 0.04 | 0.047 |
| F-1020 | NO | 2DL5_CNV | 29 | 677 | 587 | 14259 | 15552 | 0.045 | 0.04 |
| F-1021 | NO | 2DL5_CNV | 185 | 1173 | 1704 | 11394 | 14456 | 0.094 | 0.131 |
| F-1022 | NO | 2DL5_CNV | 168 | 1579 | 1304 | 11398 | 14449 | 0.121 | 0.102 |
| F-1031 | NO | 2DL5_CNV | 35 | 576 | 639 | 13100 | 14350 | 0.043 | 0.047 |
| F-1032 | NO | 2DL5_CNV | 198 | 850 | 2625 | 11220 | 14893 | 0.07 | 0.19 |
| F-1264 | NO | 2DL5_CNV | 3 | 123 | 201 | 13359 | 13686 | 0.009 | 0.015 |
| F-1266 | NO | 2DL5_CNV | 6 | 232 | 289 | 13424 | 13951 | 0.017 | 0.021 |
| F-1267 | NO | 2DL5_CNV | 0 | 27 | 88 | 13080 | 13195 | 0.002 | 0.007 |
| F-1268 | NO | 2DL5_CNV | 0 | 43 | 318 | 13443 | 13804 | 0.003 | 0.023 |
| F-1269 | NO | 2DL5_CNV | 0 | 0 | 450 | 12999 | 13449 | 0 | 0.033 |
| F-1270 | NO | 2DL5_CNV | 12 | 249 | 633 | 12725 | 13619 | 0.019 | 0.047 |
| F-1279 | NO | 2DL5_CNV | 42 | 537 | 1201 | 12472 | 14252 | 0.041 | 0.087 |
| F-1363 | NO | 2DL5_CNV | 43 | 702 | 813 | 13062 | 14620 | 0.051 | 0.059 |
| F-1364 | NO | 2DL5_CNV | 92 | 675 | 1892 | 12174 | 14833 | 0.052 | 0.134 |
| F-1399 | NO | 2DL5_CNV | 85 | 768 | 974 | 11281 | 13108 | 0.065 | 0.081 |
| F-1401 | NO | 2DL5_CNV | 17 | 295 | 757 | 13919 | 14988 | 0.021 | 0.052 |
| F-1402 | NO | 2DL5_CNV | 102 | 700 | 1806 | 12359 | 14967 | 0.054 | 0.127 |
| F-1433 | NO | 2DL5_CNV | 14 | 288 | 857 | 13923 | 15082 | 0.02 | 0.058 |
| F-1434 | NO | 2DL5_CNV | 103 | 634 | 1784 | 11376 | 13897 | 0.053 | 0.136 |
| F-1436 | NO | 2DL5_CNV | 6 | 225 | 305 | 14399 | 14935 | 0.015 | 0.021 |
| F-1438 | NO | 2DL5_CNV | 23 | 340 | 1097 | 11796 | 13256 | 0.027 | 0.084 |

**CNV analysis in other KIR genes.**

Specimens for CNV analysis in genes other than *KIR2DL5* were genomic DNA samples obtained from a number of sources. Five specimens were obtained locally. Thirteen were obtained from CEPH pedigrees, which are available from Coriell Cell Repositories.

Reference KIR CNV estimates were obtained from conventional qPCR using methods described in a previous publication (Jiang et al., 2012). qPCR reactions were carried out in quadruplicate and estimates were rounded to the nearest integer values.

We assayed eight KIR genes for which ddPCR compatible (i.e. FAM labelled) probes had been used in a previous study and which were validated for use in analogue qPCR based KIR CNV enumeration (Jiang et al., 2012). The KIR genes that were studied were *KIR2DL1*, *KIR3DL1*, *KIR2DS1*, *KIR2DS2*, *KIR2DS3*, *KIR2DS5*, *KIR3DS1* and *KIR3DP1*. In each case a single KIR gene was assayed against the single copy per haplotype *RPP30* gene.

**Figure S1-S8. Results of ddPCR KIR CNV assays.** Round points indicate ddPCR estimates, grey lines indicate qPCR estimates.

**Figure S1**


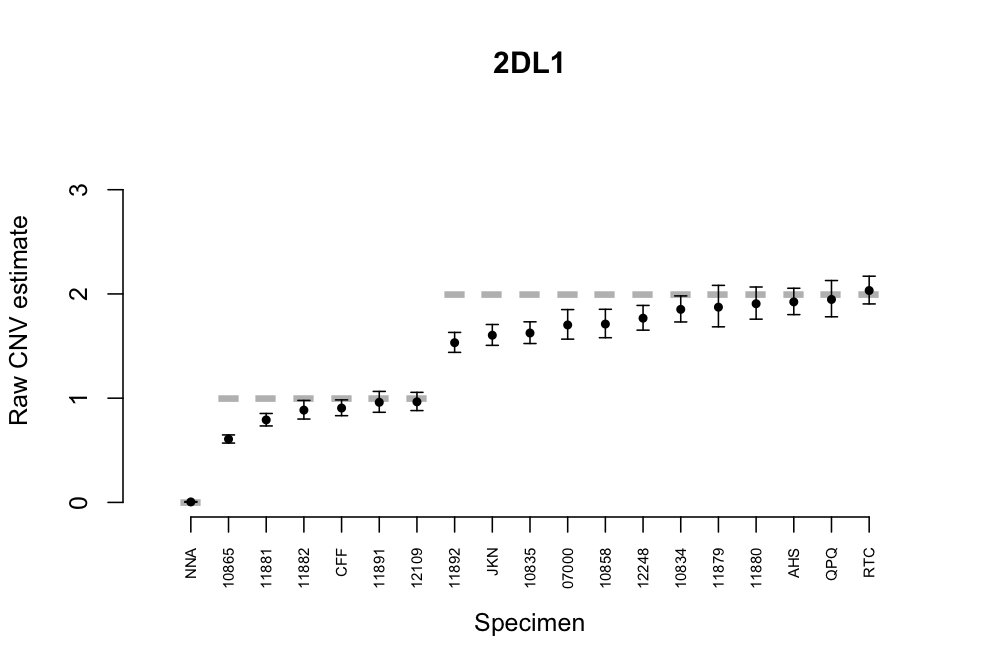


**Figure S2**


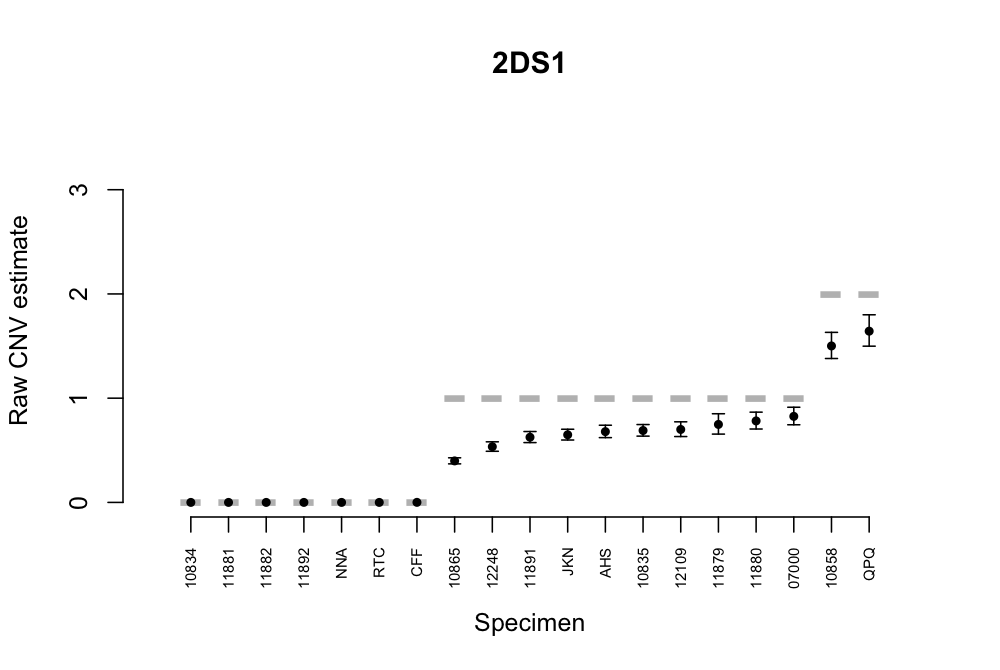


**Figure S3**


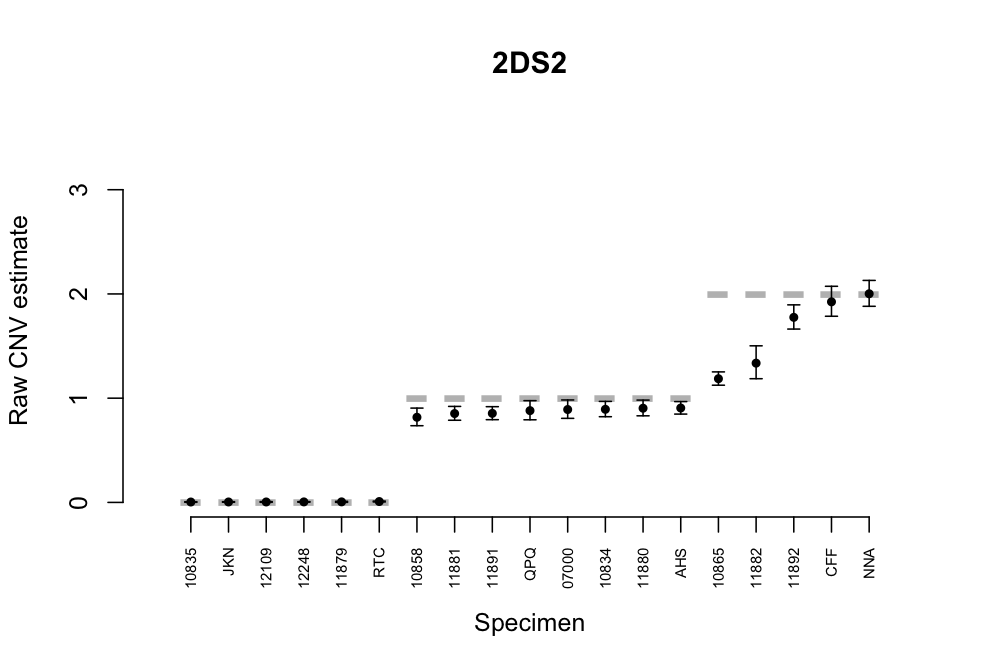


**Figure S4**


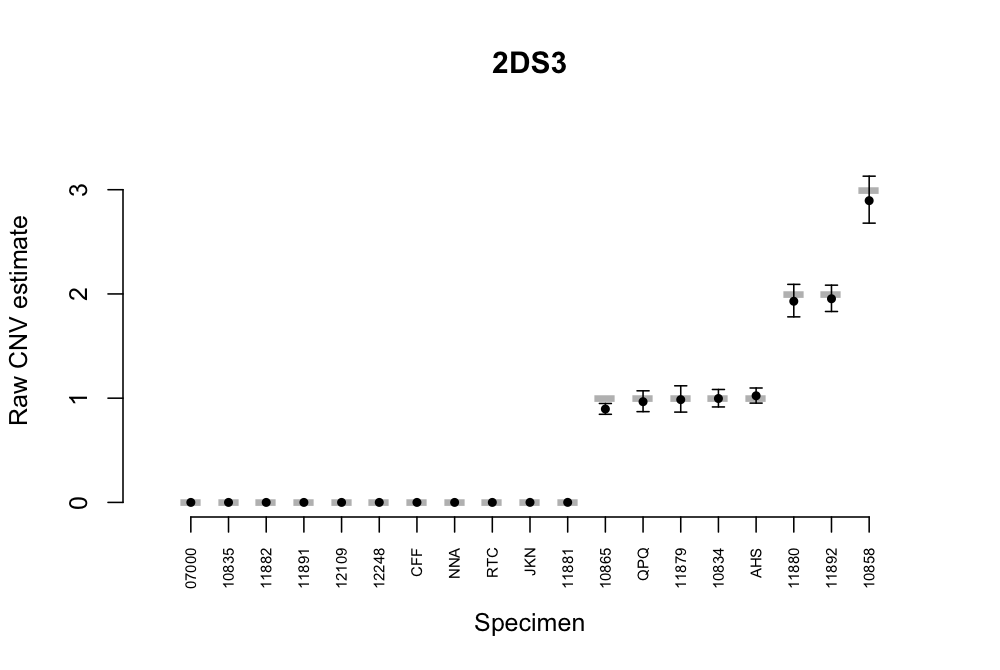


**Figure S5**


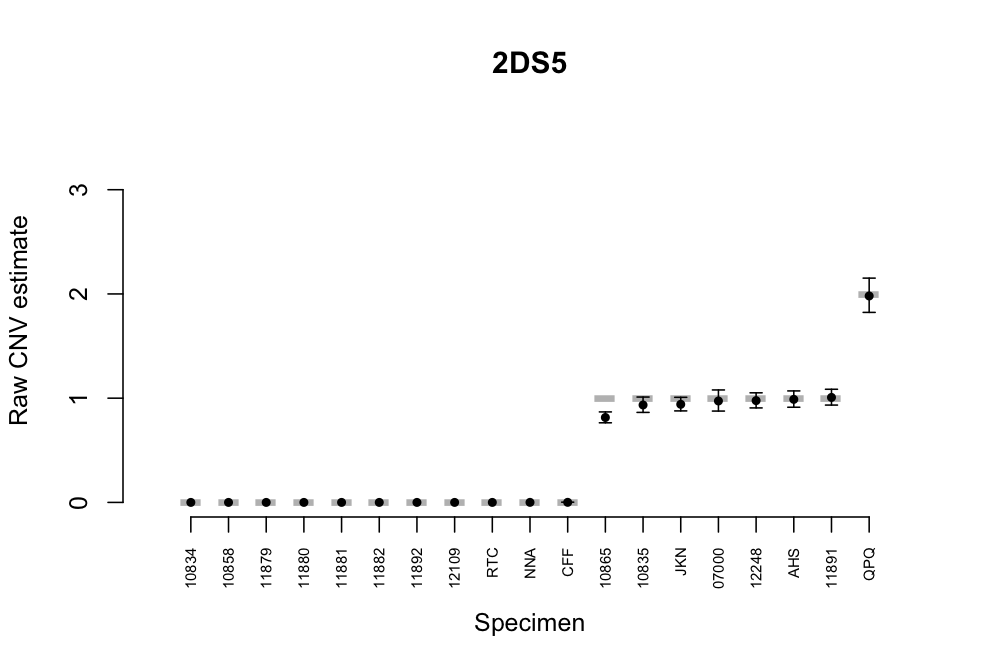


**Figure S6**


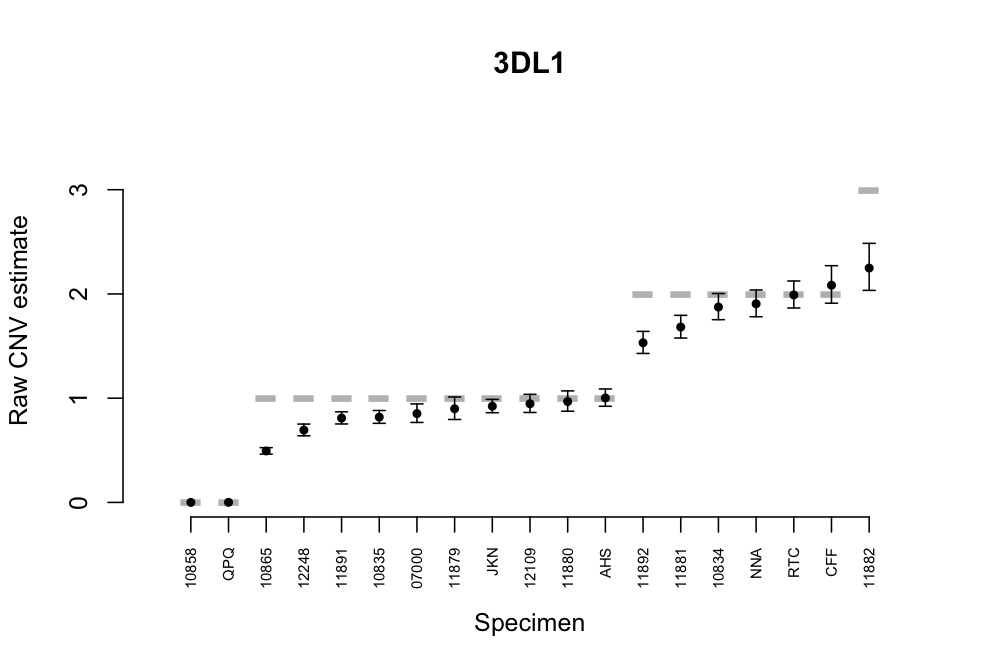


**Figure S7**


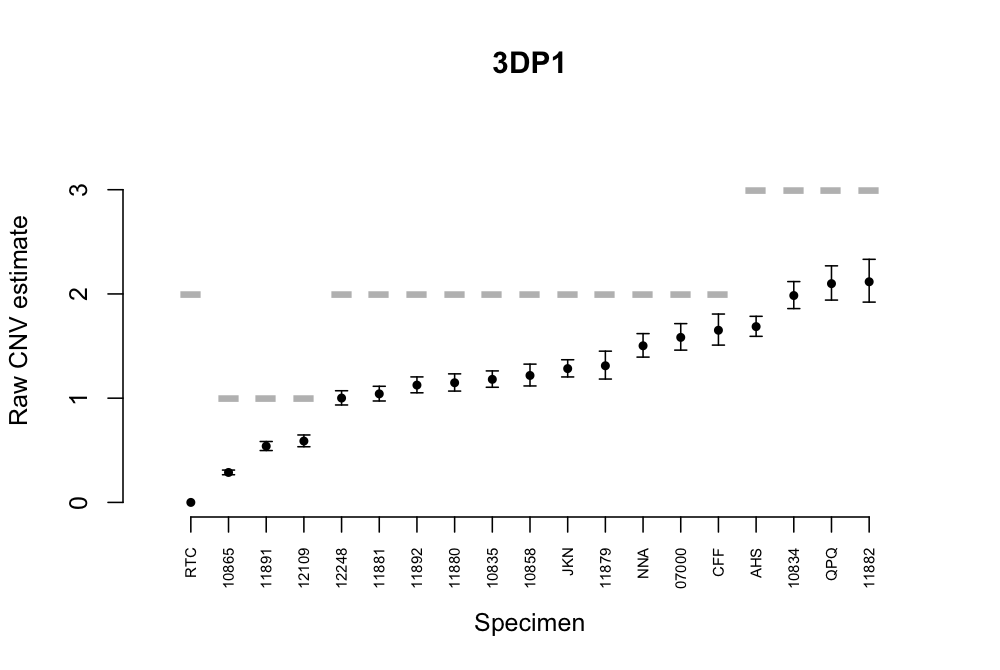


N.B. KIR3DP1 : Sample RTC repeatedly failed to amplify 3DP1 by ddPCR.

**Figure S8**


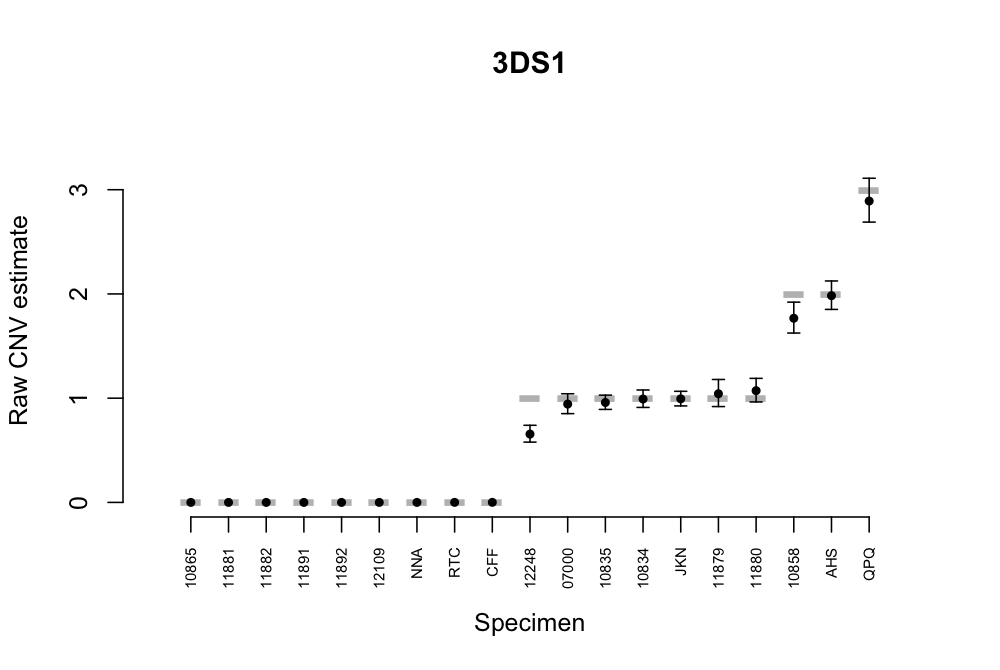


**Figure S9 : Representative droplet fluorescence intensity (FI) data.** Each point represents the FI on FAM and HEX channels for a single droplet. The following sample types are represented, (A) *KIR2DL5*^+^ *RPP30*^+^ (B) *KIR2DL5*^-^ *RPP30*^+^ (C) *KIR2DL2~KIR2DL5* linkage (higher frequency of *KIR2DL2^+^KIR2DL5^+^* double positive droplets) (D) the same sample shown in C, following restriction endonuclease digestion with diminished linkage (observable as the reduction in the frequency of *KIR2DL2^+^KIR2DL5^+^* double positive droplets (green).

**(A)**


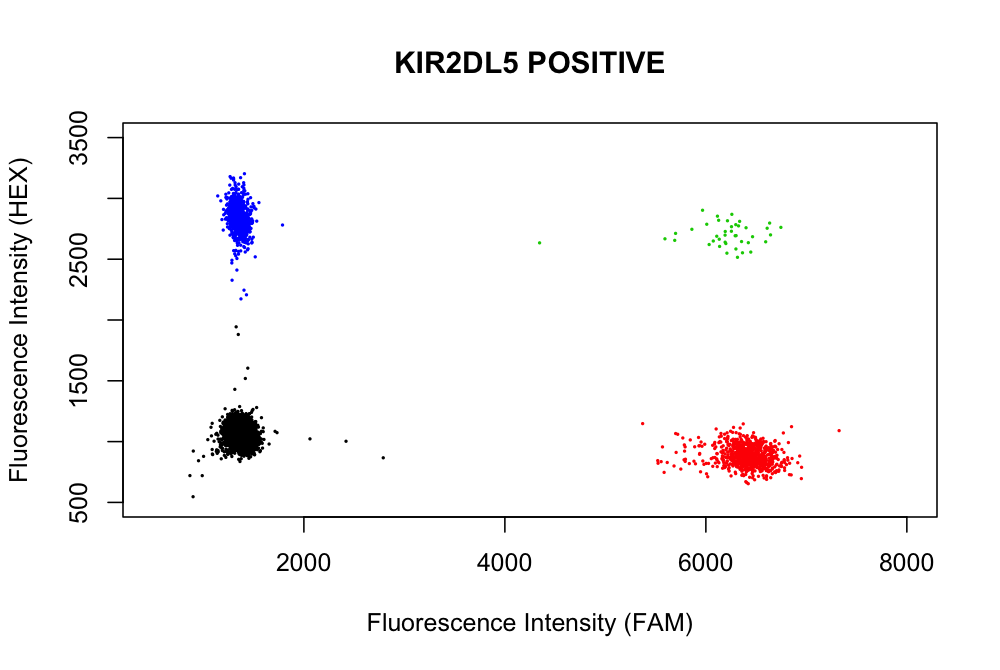


**(B)**


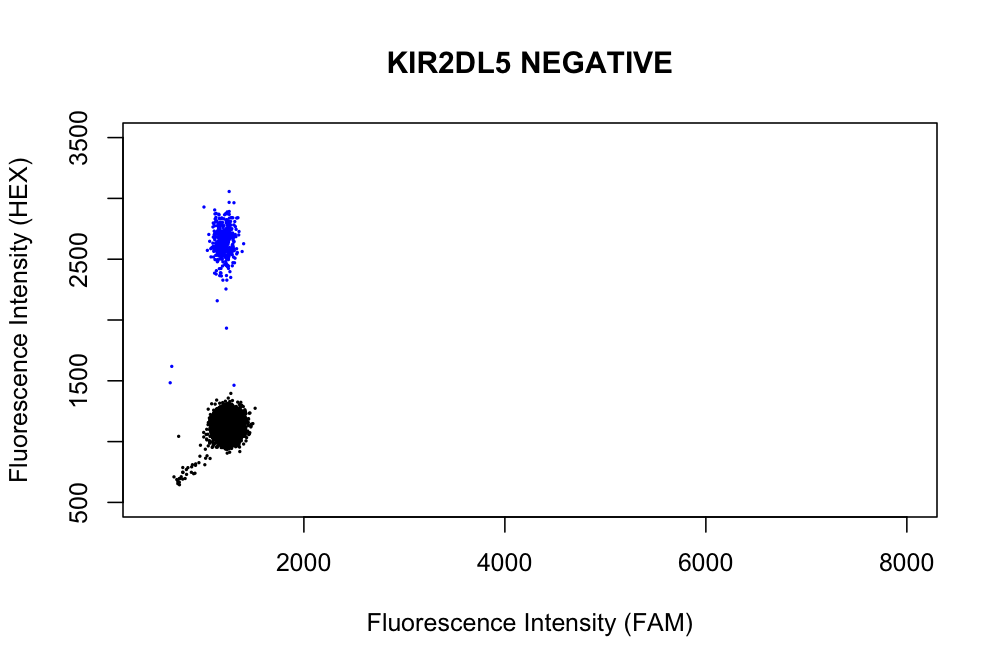


**(C)**

**
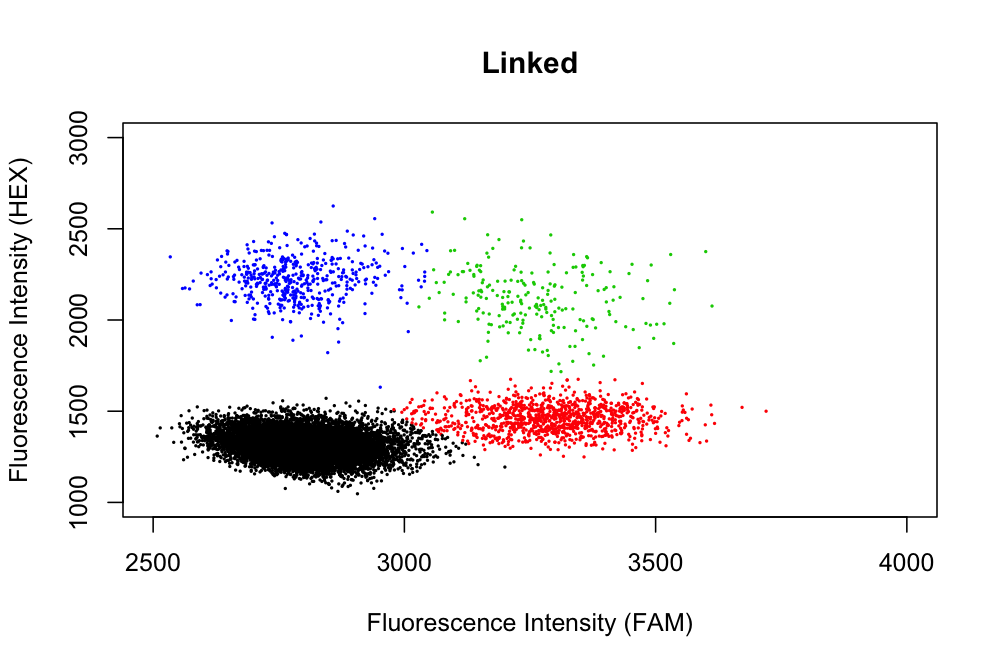
**

(D)


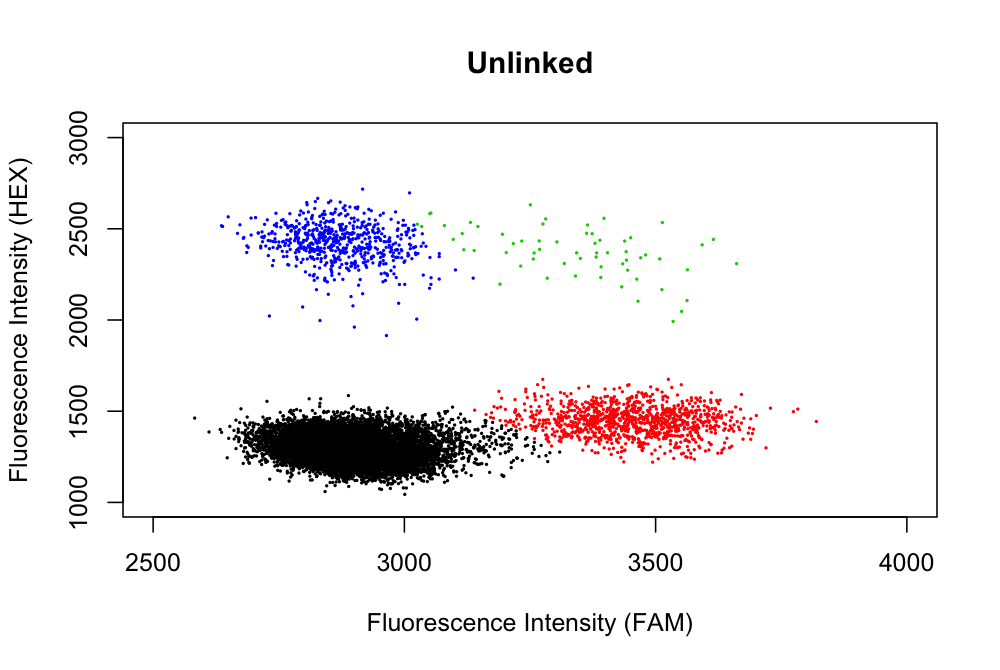


References:

Huggett, J. F., Foy, C. A., Benes, V., Emslie, K., Garson, J. A., Haynes, R., … Bustin, S. A. (2013). The digital MIQE guidelines: Minimum Information for Publication of Quantitative Digital PCR Experiments. *Clinical chemistry*, *59*(6), 892–902. doi:10.1373/clinchem.2013.206375

Jiang, W., Johnson, C., Jayaraman, J., Simecek, N., Noble, J., Moffatt, M. F., … Traherne, J. a. (2012). Copy number variation leads to considerable diversity for B but not A haplotypes of the human KIR genes encoding NK cell receptors. *Genome research*, *22*(10), 1845–54. doi:10.1101/gr.137976.112
